# Development of thrombocytopenia is associated with improved survival in patients treated with immunotherapy

**DOI:** 10.2144/fsoa-2020-0021

**Published:** 2020-06-25

**Authors:** Hussein A Assi, Adam S Asch, Michael Machiorlatti, Sara K Vesely, Sami Ibrahimi

**Affiliations:** 1 Division of Hematology/Oncology, Stephenson Cancer Center, University of Oklahoma Health Sciences Center, Oklahoma City, OK 73104, USA; 2 Department of Biostatistics & Epidemiology, Hudson College of Public Health, University of Oklahoma Health Sciences Center, Oklahoma City, OK 73104, USA

**Keywords:** checkpoint inhibitors, immune-mediated, immunotherapy, PD1/PDL-1 inhibitors, platelets, prognosis, survival, thrombocytopenia

## Abstract

**Background:**

Immune-related adverse events are associated with efficacy of immune checkpoint inhibitors (ICIs). We hypothesize that immune-mediated thrombocytopenia could be a biomarker for response to ICIs.

**Materials & methods:**

This retrospective study included 215 patients with metastatic malignancies treated with ICIs. Patients were stratified by nadir platelet count. Outcomes of interest were progression-free survival and overall survival.

**Results:**

On multivariate analysis, grade 1 thrombocytopenia was positively associated with overall survival compared with patients who did not develop thrombocytopenia (hazard ratio [HR]= 0.28 [95% CI: 0.13–0.60]; p = 0.001), while grade 2–4 thrombocytopenia was not (HR= 0.36 [95% CI: 0.13–1.04]; p = 0.060). There was no association between degree of thrombocytopenia and progression-free survival.

**Conclusion:**

Follow-up studies are warranted to substantiate the predictive significance of thrombocytopenia in patients receiving ICIs.

## Lay abstract

Immune checkpoint inhibitors (ICIs) are a class of drug that are increasingly being used in different cancers. The extent of response to treatment with ICIs differs among individuals. There is a lack of biomarkers, which would help clinicians predict response to ICIs. In our study, we aimed to explore the development of low platelets as a potential biomarker. Our findings suggest that cancer patients receiving ICIs who develop a mild decrease in platelet count tend to live longer than those who do not. However, this was not true for those who developed a severe decrease in platelet count. The mechanism of how ICIs affect platelets may be related to the interaction between the immune system and platelets. More studies are needed to validate our results and better understand the role of platelets in cancer biology.

Cancer immunotherapy modulates the host’s tumor-directed immune response and has been an effective strategy in treating various tumors. Specifically, CTLA-4, PD-1 and its associated ligand (PD-L1) have been three of the most targeted checkpoints, effectively overcoming tumor’s immune escape mechanisms in a large subset of patients [1]. In fact, the number of clinical trials using PD1/PDL-1 inhibitors has increased over 600% from 2015 to 2017 alone [2]. Moreover, the estimated number of cancer patients eligible for immune checkpoint inhibitors (ICIs) increased from 1.5% in 2011 to 43.6% in 2018 [2]. Despite the increased use and efficacy of ICIs across a wide range of malignancies, a large proportion of patients do not seem to derive any benefit from treatment [3]. Thus, there has been extensive attempts at identifying clinical, cellular and molecular predictive biomarkers to guide treatment recommendation [4–7]. However, only few blood biomarkers have been studied, including baseline neutrophil-to-lymphocyte ratio and LDH levels [8,9]. Thrombocytopenia has been reported in approximately 15–37% of patients receiving ICI therapy [10,11]. The precise pathogenesis of this immune-mediated thrombocytopenia is not entirely understood [12]. Other immune-related adverse events (irAEs), mostly endocrine, gastrointestinal and dermal in nature, have been shown to be associated with the efficacy of PD-1/PD-L1 inhibitors in patients with melanoma and non-small-cell lung cancer [13–15]. However, this association has not been studied with thrombocytopenia. This study aims to evaluate whether the development of thrombocytopenia after ICI therapy is associated with disease and survival outcomes.

## Materials & methods

### Study design

We conducted a retrospective study of patients aged 18 years and older with various malignancies treated with ICIs in the metastatic setting between January 2014 and January 2016 at the University of Oklahoma Health Sciences Center. Patients did not receive concurrent chemotherapy with the ICI; however, patients did receive various chemotherapies prior and/or after ICI therapy. Patient’s clinicopathologic characteristics included age, gender, race, cancer type, Eastern Cooperative Oncology Group Performance Status, ICI therapy agent and line of therapy. Baseline (within 30 days of ICI therapy initiation) and nadir platelet counts (up to 6 months after ICI therapy) were obtained. Platelet count was reported in conventional units (×10^3^/μl of blood). Patients were grouped according to their nadir platelet count: normal platelets (≥150), grade 1 (75–150) and combined grades 2, 3 and 4 (<75). Follow-up time was calculated from the date of first ICI therapy administration to the last date of contact. Progression-free survival (PFS) was defined as the number of months between date of first ICI administration and date of first disease progression, death or last follow-up. Overall survival (OS) was defined as the number of months between date of first ICI administration and date of death or last follow-up. All procedures followed were in accordance with the ethical standards of the responsible committee on human experimentation (institutional and national) and with the Helsinki Declaration of 1975, as revised in 2008 (5). The objective of this study is to assess whether the degree of thrombocytopenia is associated with PFS and OS in patients receiving ICI therapy.

### Statistical analysis

Patient’s clinicopathologic characteristics were analyzed using simple descriptive statistics (median [range] for continuous covariates and n [%] for categorical variables). Univariate and multivariable Cox proportional hazard regression models were used to assess the association of thrombocytopenia with PFS and OS. Hazard ratios with corresponding 95% CIs were reported, while p-values <0.05 were considered to be significant. Proportionality assumptions were assessed. Kaplan–Meier survival curves were created for PFS and OS categorized by nadir platelet count. SAS version 9.4 was used to perform all data analysis.

## Results

### Patient characteristics

A total of 250 patients with metastatic cancers treated with ICIs were screened. After excluding 35 patients with baseline platelet count <150, 215 patients were included in the analysis. Median age at diagnosis was 62 (range: 23–91) years, with 54% females. The most common cancer diagnoses were lung cancer (27%), melanoma (19%) and gynecologic cancer (15%). Pembrolizumab was the most commonly used agent (38%), followed by Nivolumab (32%). Median number of treatment cycles received was four (range 1–15). Most patients (81.9%) had previously received at least one line of therapy prior to receiving ICI. None of the patients received any platelet transfusions during the 30 days prior to ICI initiation. A summary of baseline characteristics of patients are listed in Table 1. Out of 215 patients, 202 patients had complete platelet data and were therefore included in the PFS and OS analysis. Median platelet count at baseline and nadir were 237 (152–535) and 183 (4–552), respectively. Median time to nadir platelet count was 54 days (2–823). Thrombocytopenia of any grade was observed in 62 (31%) patients. The distribution of patients among thrombocytopenia grades is listed in Table 2. Median follow-up time was 226 days. Out of 14 patients with grade ≥2 thrombocytopenia, 5 (36%) received platelet transfusions, two of whom had a bleeding event. Immunotherapy was held for all patients with grade 3 and 4 thrombocytopenia.

**Table 1.** Descriptive characteristics of patients.

Characteristics	All patients (n = 215)
Age (years) Median (range)	62 (23–91)
Gender, n (%) – Male – Female	99 (46.0) 116 (54.0)
Race, n (%) – Whites – Blacks – Hispanics – Others	188 (87.5) 17 (7.9) 5 (2.3) 5 (2.3)
Cancer type, n (%) – Lung – Melanoma – Urothelial – Renal – Head and neck – Gynecologic – Other	59 (27.4) 40 (18.6) 15 (7.0) 14 (6.5) 24 (11.2) 33 (15.3) 30 (14.0)
ECOG PS, n (%) – 0 – 1 – 2/3	63 (29.3) 115 (53.5) 37 (17.2)
Checkpoint inhibitor, n (%) – Nivolumab – Pembrolizumab – Ipilimumab/nivolumab – Atezolizumab – Other	68 (31.6) 81 (37.7) 20 (9.3) 14 (6.5) 32 (14.9)
Line of therapy, n (%) – 1 – 2 – ≥3	39 (18.1) 94 (43.7) 82 (38.1)

ECOG PS: Eastern Cooperative Oncology Group Performance Status.

**Table 2.** Platelet count at baseline and follow-up of all patients.

Variable	All patients (n = 215)
Baseline platelet count (x10^3^/μl) – Median (range)	237 (152–535)
Nadir platelet count (x10^3^/μl) – Median (range)	183 (4–552)
Nadir platelet count, n (%) – ≥150k – 75k–150k – <75k	140 (69.3) 48 (23.8) 14 (6.9)
Time to nadir (days) – Median (range)	54 (2–823)

### Survival analysis by thrombocytopenia grade

**Figure 1.**
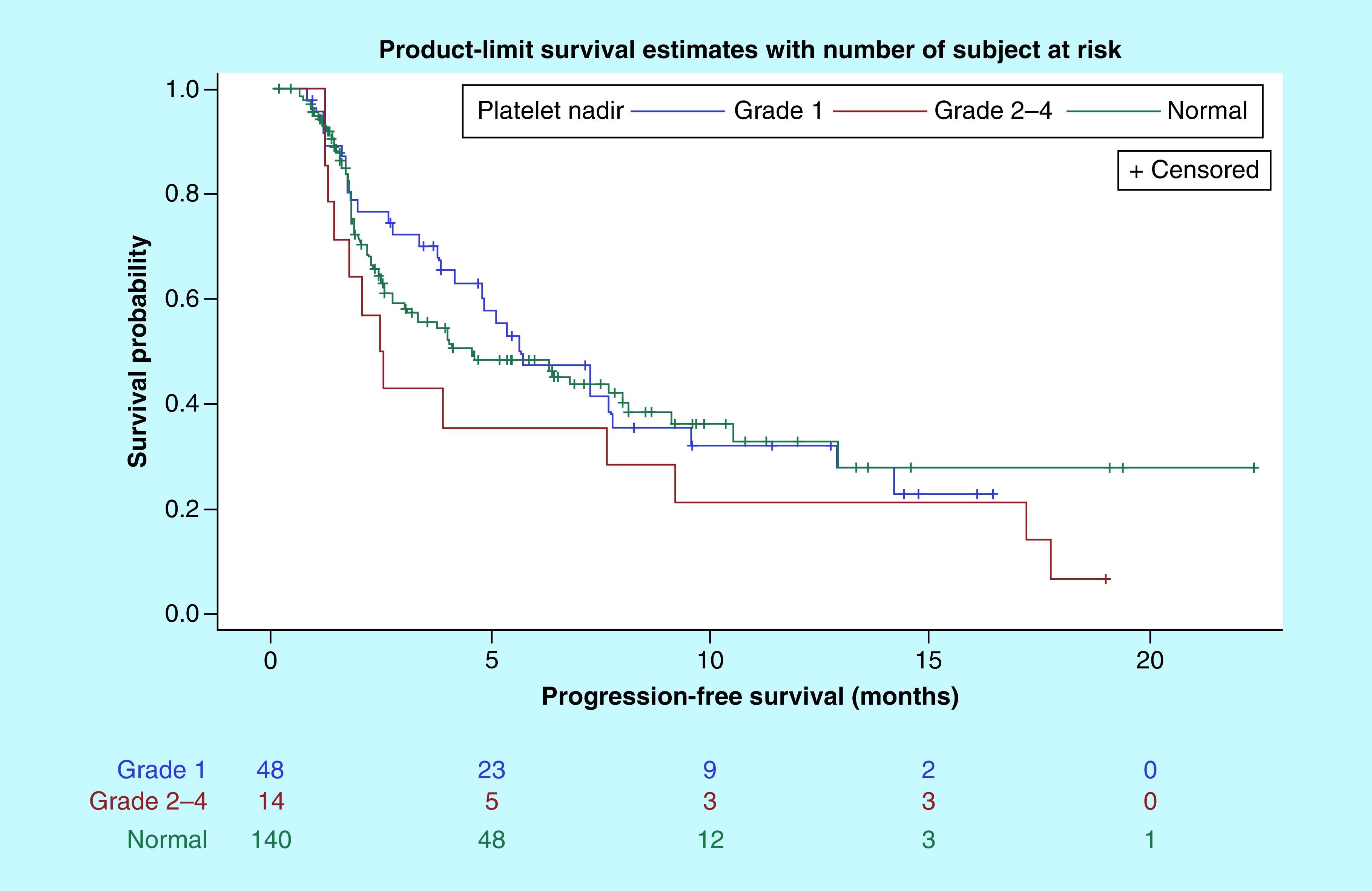
Kaplan–Meier curves of progression-free survival by thrombocytopenia group. PFS: Progression-free survival.

**Figure 2.**
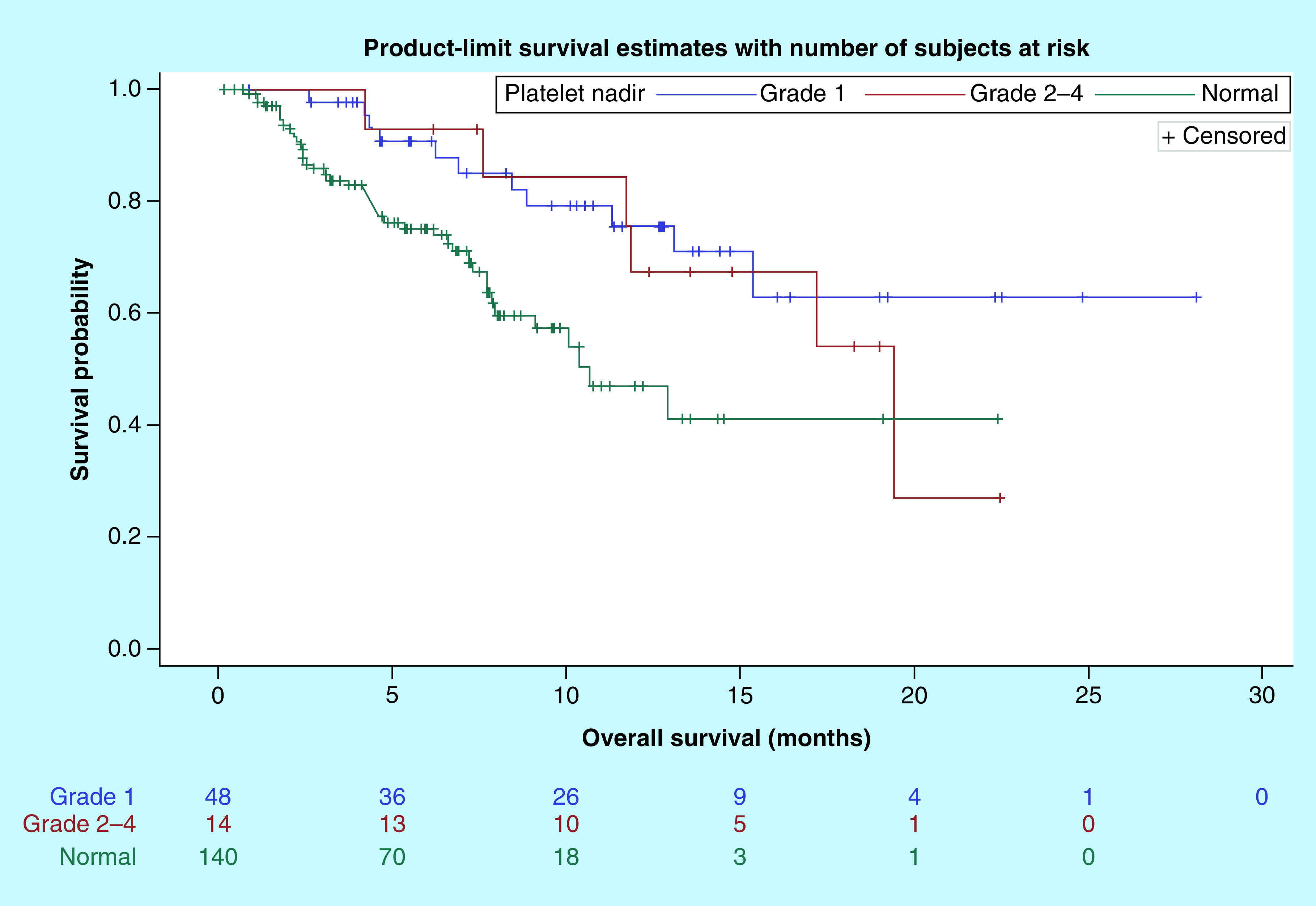
Kaplan–Meier curves of overall survival by thrombocytopenia group. OS: Overall survival.

Kaplan–Meier curves of PFS and OS by thrombocytopenia group are shown in Figure 1 & Figure 2, respectively. The median PFS for patients with normal nadir platelet count, grade 1 thrombocytopenia; grade 2–4 thrombocytopenia was 4.6 months (95% CI: 2.8–8.0), 5.7 months (95% CI: 3.9–9.5) and 2.5 months (95% CI: 1.3–9.2), respectively. The median OS was not reached (NR) for patients who developed grade 1 thrombocytopenia, compared with 10.6 months (95% CI: 7.9–NR) and 19.4 months (95% CI: 11.7–NR) in patients with normal nadir platelet count and grade 2–4 thrombocytopenia, respectively. Cox univariate analysis and multivariate analysis of PFS and OS, adjusted for age, gender, race, cancer type, performance status, ICI type and line of therapy, are shown in in Table 3 & Table 4, respectively. There was no difference in PFS between the three groups. There was a 72% reduction in hazard of death for patients with grade 1 thrombocytopenia compared with those who did not develop thrombocytopenia. However, there was no significant difference in OS between patients with grade 2–4 thrombocytopenia and normal platelets (hazard ratio = 0.36 [95% CI: 0.13–1.04]; p = 0.060).

**Table 3.** Univariate and multivariate Cox proportional hazards model analysis of progression-free survival (n = 202).

Parameter	Univariate analysis			Multivariable analysis^†^		
	HR^‡^	95% CI^§^	p-value	HR^‡^	95% CI^§^	p-value
Nadir platelet count – Normal – Grade 1 – Grade 2–4	0.95 1.47	Reference 0.62–1.46 0.80–2.69	0.813 0.213	0.70 1.02	Reference 0.44–1.13 0.49–2.12	0.144 0.953

† Adjusted for age, race, cancer type, performance status, immune checkpoint inhibitor type and line of therapy.

‡ HR: Hazard ratio.

§ CI

**Table 4.** Univariate and multivariate Cox proportional hazards model analysis of overall survival (n = 202).

Parameter	Univariate analysis			Multivariable analysis^†^		
	HR^‡^	95% CI^§^	p-value	HR^‡^	95% CI^§^	p-value
Nadir platelet count – Normal – Grade 1 – Grade 2–4	0.37 0.52	Reference 0.18–0.73 0.21–1.25	0.004 0.143	0.28 0.36	Reference 0.13–0.60 0.13–1.04	0.001 0.060

† Adjusted for age, race, cancer type, performance status, immune checkpoint inhibitor type and line of therapy.

‡ HR: Hazard ratio.

§ CI.

## Discussion

Adverse events from systemic therapy affect patients’ quality of life and often require dose reduction or even treatment discontinuation. Occurrence of side effects from therapy has been demonstrated to be an independent prognostic indicator of response, even in the preimmunotherapy era [16]. A study of fluoropyrimidine monotherapy in advanced gastrointestinal cancers showed response rates of 22.7, 8.7 and 15% for patients who developed mild–moderate leukopenia, no leukopenia and severe leukopenia, respectively [16,17]. Even with targeted therapies, skin rash and hypertension was found to be positively correlated with improved PFS and OS for EGFR antibodies and VEGF inhibitors, respectively [18]. The repertoire of irAEs reported in immunotherapy clinical trials has been expanding. In a study of non-small-cell lung cancer, development of any irAEs with Nivolumab was associated with superior disease and survival outcomes [19]. The most common irAEs included skin rash (25%), gastrointestinal toxicities (9%) and endocrinopathies (8%) [19]. Although immune-related hematologic toxicities are not as common, they may also indicate efficacy of ICIs. One prospective study involving advanced melanoma patients demonstrated that an increase in eosinophil count by >100/mm^3^ or absolute lymphocyte count by >200/mm^3^ between the first and second Ipilimumab infusion were associated with improved OS [20]. Another retrospective study determined that lymphopenia is associated with a trend in improved PFS, but this was not statistically significant [21]. Thrombocytopenia would be particularly interesting to investigate, as the immune functions of platelets are being unraveled.

Blood platelets have traditionally been known for their role in hemostasis and thrombosis [22]. Furthermore, platelets have been implicated in tumorigenesis and tumor metastasis [23]. There are many proposed mechanisms that have been suggested to explain this intriguing role of platelets. Platelet α-granules are enriched in growth factors, including TGF-β, EGF and PDGF. Upon platelet activation, the release of these growth factors leads to potentiation of cellular proliferation, growth and differentiation [22]. Moreover, it is theorized that platelets may undergo mRNA splice events in response to signals released by tumor cells [23]. Another suggested mechanism includes sequestration of tumor-associated molecules into the platelets, leading to what is known as tumor-educated platelets [24]. Based on these findings, it has become clear that interactions between cancer cells and activated platelets occur at different cellular levels; it may be a potential target for future therapeutic drugs. Nevertheless, more studies are needed to better delineate the role of platelets in cancer biology.

Another finding that further supports the role of platelets in cancer immunotherapy is the detection of PD-L1 expression on platelets. A recent study by Rolfes *et al.* demonstrated that PD-L1 is preferentially expressed on platelets of patients with head and neck squamous cell carcinoma compared with healthy patients [25]. In fact, most healthy donor platelets were negative for PD-L1 by flow cytometry. Moreover, the amount of PD-L1-expressing platelets diminished in the blood of four lung cancer patients treated with atezolizumab, a PD-L1 inhibitor. Interestingly, the total platelet count was not affected [25]. While this mechanism does not explain the thrombocytopenia seen in our cohort of patients, it raises the possibility that platelets may play a role in modulating immune response to cancer.

Our study supports the notion that grade 1 thrombocytopenia during treatment with ICIs is positively associated with OS, when compared with those who do not develop thrombocytopenia. This survival advantage was not seen with higher grades of thrombocytopenia. Interestingly, the superior OS in grade 1 thrombocytopenia was seen despite the lack of PFS benefit. This is commonly seen in studies involving ICIs, as the traditional response evaluation criteria in solid tumors may not accurately depict PFS in this group of patients, such as in cases of pseudo-progression [26]. Moreover, ICIs can have prolonged, durable responses in a subgroup of patients even after discontinuation of treatment, which can skew the OS benefit beyond that seen with PFS [26]. Nonetheless, the development of thrombocytopenia as an immune-mediated reaction may serve as an indicator of prolonged survival in patients treated with ICI therapy.

Our study has several limitations. The retrospective nature of this study makes it impossible to derive causation between ICI administration and thrombocytopenia. Data on clinical or radiographic response to ICI therapy, the exact number of cycles between ICI initiation and the start of thrombocytopenia, as well as information on concomitant organ toxicities and use of immunosupressants were missing. Moreover, physiological fluctuations in platelet count are difficult to account for and may confound the results. In addition, only a small sample of patients had higher grades of thrombocytopenia. Therefore, validation of these results in different malignancies and ICI types in a prospective fashion or a larger retrospective study is warranted. At the cellular level, our findings raise even more questions about the physiologic effect of immunotherapy on platelets, both quantitatively as well as qualitatively. The predictive significance of the development of thrombocytopenia in patients receiving ICI therapy warrants further investigation.

## Future perspective

As immunotherapy is increasingly being incorporated into the treatment plan of patients with various malignancies, predictive biomarkers of response to ICIs are urgently needed for appropriate patient selection. The role of platelets in cancer immunology is becoming more apparent. Interestingly, the recent finding of PD-L1 expression on platelets further supports this role, although the exact function of platelet PD-L1 is currently unknown. This novel discovery lays the ground for additional experiments exploring the cancer cell–platelet interaction. Liquid biopsies, specifically analyzing platelets and their level of PD-L1 expression, could potentially be used in the clinical setting to predict response to ICIs. Furthermore, these liquid biopsies looking at platelets could be assessed for potential correlation with clinical response in patients receiving immunotherapy. Cancer immunology is a rapidly evolving field with a promising future in addressing current unmet needs, specifically the lack of biomarkers for immunotherapy.

**Summary points**

- Immune-related adverse events have been demonstrated to be associated with the efficacy of immune checkpoint inhibitors (ICIs) in various malignancies.
- Although immune-related hematologic toxicities are not common, they may also indicate efficacy of ICIs.
- Platelets have been shown to have a role in cancer immunotherapy.
- The objective of this study was to evaluate whether the development of thrombocytopenia after ICI therapy is associated with disease and survival outcomes.
- We conducted a retrospective study of 215 adult patients with various malignancies treated with ICIs in the metastatic setting between January 2014 and January 2016 at the University of Oklahoma Health Sciences Center.
- Our study suggests that in patients treated with ICIs, grade 1 thrombocytopenia is positively associated with overall survival.
- Grade 2–4 thrombocytopenia was not associated with superior overall survival.
- There was no association between degree of thrombocytopenia and progression free-survival.
- Immune-mediated thrombocytopenia could be a biomarker for response to ICIs.
- Follow-up studies to confirm our findings are warranted to substantiate the predictive significance of thrombocytopenia in patients receiving ICIs.
